# Simulation of the impacts of high temperature stress on pepper (*Capsicum annum* L.) yields

**DOI:** 10.3389/fpls.2025.1590193

**Published:** 2025-05-26

**Authors:** Bomi Park, Sojung Kim, Sumin Kim

**Affiliations:** ^1^ Department of Environmental Horticulture & Landscape Architecture, College of Life Science & Biotechnology, Dankook University, Dongnamgu, Cheonan-si, Chungnam, Republic of Korea; ^2^ Industrial and Systems Engineering, Dongguk University-Seoul, Seoul, Republic of Korea

**Keywords:** heat stress, process-based model, pepper, morphological traits, nitrogen analysis

## Abstract

Improving the accuracy of yield predictions for cash crops such as pepper (*Capsicum annum* L.) has increasingly captured the interest of many scientists in South Korea. This study marks the first initiative to develop yield prediction tools for peppers cultivated under heat stress conditions. To refine the yield prediction model, field studies were conducted to establish the plant growth curve and parameter sets for two different pepper accessions, PHR18 and PHR23, under heat stress conditions. According to field studies, the two pepper accessions exhibited distinct growth patterns under prolonged heat stress conditions. PHR18 experienced significant heat stress effects in the first month of exposure, whereas it demonstrated stress priming to regain growth by the 75^th^ day of heat stress exposure. PHR23, having a larger leaf area, accumulated more biomass than fruit yields in the initial month of exposure, thus increasing its yields at higher temperature conditions due to enhanced photosynthesis rates. The crop growth curve and parameters were formulated based on these studies, and the open field simulations were calibrated with measured yields from multiple locations in South Korea from 2020-2024. Consequently, a robust pepper growth model was developed and employed to assess the effects of heat stress on the yields of two pepper accessions across various South Korean locations. The development of this crop growth model under stressful conditions will aid farmers and policymakers in making informed decisions during extreme events.

## 1 Introduction

Global warming and rising temperatures have been negatively impacting summer vegetable production. Higher temperatures can lead to heat stress, disrupting plant growth processes and reducing yield. According to reports of Intergovernmental Panel on Climate Change (IPCC, 2023), the global temperature has been already increased by 1.1°C, and every 0.5°C of global temperature rise will causes increases in frequency and severity of heat extremes. The summer 2023 has been recorded as the hottest summer since global record began in 1880 (NASA, 2023). South Korea has also experienced its hottest summer on record in 2023. According to the Korea Meteorological administration (KMA), the average temperature from June to August was 25.6°C, which is hottest summer temperature since 1973 (KMA, 2024). These increased temperatures pose a significant threat to farmers and agricultural productivity.

Prolonged periods of abnormally high temperatures can seriously affect crop growth, development, physiological processes, and yield (Moore et al., 2021). High temperatures can scorch twigs and leaves, cause visual symptoms of sunburn on stems, leaves, and branches, premature senescence and abscission of leaves, inhibit shoot and root growth, and result in discoloration and damage to fruits (Wahid et al., 2007; Lipiec et al., 2013; Hatfield and Prueger, 2015). Heat stress may also reduce cell division and limit cell elongation, consequently retarding plant growth (Ashraf and Harris, 2004; Camejo et al., 2005; Daly et al., 2004). Furthermore, heat stress can significantly diminish photosystem activity due to a reduction in chlorophyll biosynthesis (Dutta et al., 2009). Under high temperature stress (≥ 32°C), floret sterility may increase, linked to diminished anther dehiscence, poor pollen shedding, limited pollen grain germination on the stigma, decreased pollen tube elongation and reduced pollen germination (Fahad et al., 2015, 2016). As plant responses to heat stress can vary between species or even cultivars (Barua et al., 2008; Sakamoto and Murata, 2000), they exhibit certain changes in their growth patterns and physiological processes to mitigate damage from heat stress (Fahad et al., 2017).

The sustainability to high temperatures varies among plant species and genotypes, particularly during critical stages of development such as the vegetative and reproductive phases. Heat stress-tolerant plants employ strategies such as ‘stress-priming’ to adapt, survive, and realize their full growth potential under stressful conditions (Sanyal et al., 2018). Stress priming is a process that preserves heat stress memory for several days (Charng et al., 2006, 2007; Lämke and Bäurle, 2017). Then, heatstress priming mediates memory establishment, inducing complex reprogramming of cellular mechanisms to enhance stress tolerance (Sanyal et al., 2018). This process is crucial for plants exposed to prolonged heat stress conditions. A recent study (Kim et al., 2023) reported that peppers (*Capsicum annum L.)* can be primed by heat stress. After days of heat stress priming, peppers alter their morphological characteristics (e.g., leaf area, height, stem thickness) to mitigate further effects of heat stress (e.g., drought). However, the economic yields of peppers under heat stress conditions were lower than those under control conditions. Moreover, yield damage levels varied among pepper genotypes. According to this study, understanding the physiological and morphological responses of different peppers to heat stress is vital to minimizing damage from unpredictable climates. Additionally, cropping management strategies (e.g., genotype selection, planting date) that optimize yield production under stress conditions are crucial for addressing food security issues in the context of global warming.

Pepper is one of the major spice crops consumed in South Korea, where stable crop production is closely linked to the stability of farm income and food prices. However, the continuous rise in air temperature poses a severe threat to food security in Korea. Although pepper thrives in warm climates, during hot summers, when temperatures exceed 35°C, the economic yields of pepper can significantly drop (Zakir et al., 2024). In South Korea, the number of days with air temperatures above 35°C has been rising continuously since the 1980s (Greenpeace, 2021). Consequently, developing effective cropping management to sustain pepper production under unpredictable weather conditions is essential for food security. To devise a robust cropping plan, it is crucial to understand the effects of prolonged heat stress on pepper yields across various locations and among different genotypes.

A crop growth model can be a great tool to assess the impact of abiotic stress such as heat stress on crop yields. Many modeling studies have estimated the effects of climatic variability (e.g. drought, increased temperature, etc) on crop yields (Wang et al., 2016; Zare et al., 2023). To obtain realistic simulation results, the models should be able to capture dynamics of soil, water, and other environmental factors under various climate conditions, which are strongly associated with crop yield changes. Thus, a process-based model with soil and water components were often employed. For example, Wang et al. (2016) used SWAT (Wang et al., 2016) to simulate dynamics of soil moistures and how corn responses to wet and dry soil conditions. However, there are not many studies that have simulated heat stress effects on crop yields due to limited field studies on heat stress impacts on crops. Most heat stress studies have conducted experiments monitoring heat stress responses only for relatively short growth periods or only one of growth stage periods (e.g. flowering, seedling, or germination stages), which does not provide adequate data for model development. To increase model’s accuracy, results from experiments that investigate plant responses to prolonged term heat exposure are required for model calibration and validation.

In this study, the aims of this study is to study the role of heat stress on pepper production across multiple regions in South Korea. The model was based on a 2-year field study involving two different pepper cultivars with distinct growth patterns. Unlike other major grain crops such as corn and soybean, a vegetable growth model to examine the effects of heat stress is underdeveloped. This study marks the first attempt to simulate the growth of two different pepper cultivars under heat stress conditions. To enhance simulation accuracy, key plant parameters, including the plant growth curve, were derived from 2-year field experiments predominantly conducted under heat stress treatment. The field study was conducted under controlled conditions, enabling the evaluation of physiological and morphological changes in plants exposed to heat stress. Following the development of the plant growth model, the impact of heat stress on the yields of two different pepper cultivars was assessed in multiple locations where peppers are primarily produced in Korea. These simulation results will provide valuable insights into the most suitable cropping plans for farmers, scientists, and policymakers aiming to improve crop productivity amid climate-induced stresses in South Korea.

## 2 Materials and methods

### 2.1 Plant materials, treatment, and experimental setup

The field study builds on previous research (Kim et al., 2023) by incorporating an additional year to assess the prolonged effects of heat stress on the physiological, morphological, and yield characteristics of different pepper (*C. annum* L.) accessions. For this study, only two hot pepper accessions, including PHR18(accession code of IT286261) and PHR23(commercial cultivar name of ‘Big Star’), were chosen due to their distinct morphological and physiological responses to long-term heat stress treatment. PHR18 has been known as heat sensitive accession, while PHR23 has been known as heat tolerant accession. In this study, only selected morphological and physiological characteristics of two accessions, which were employed for developing plant parameters in simulation, were analyzed.

**Figure 1:**
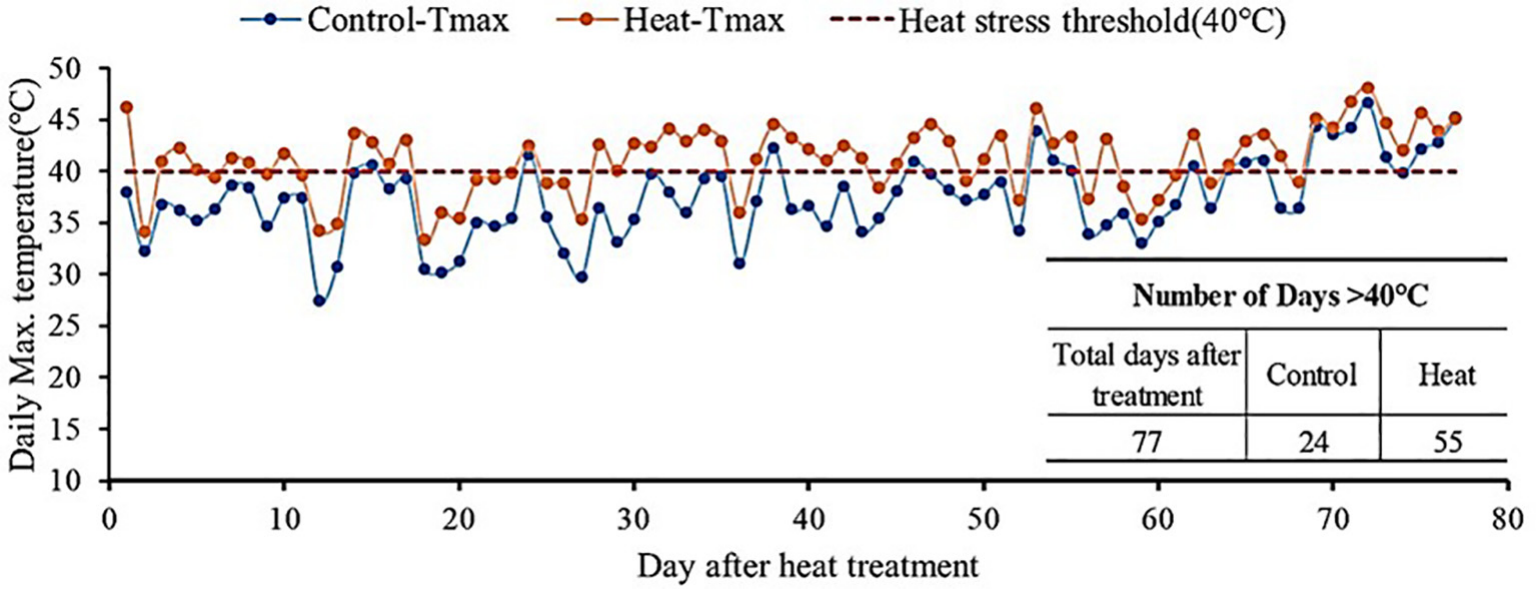
Annual average temperature in the greenhouse under control conditions and high temperature stress conditions in 2022 and 2023.

The experiment was conducted in two plastic-covered greenhouses at the National Institute of Horticultural and Herbal Science (Wanju, Korea, 35°83′ N, 127°03′ E) during 2022-2023. Daytime temperatures in the control and heat-treated greenhouses were set at 28°C and 30°C, respectively. And nighttime temperatures in the control and heat-treated greenhouses were 18°C and 22°C, respectively. **Figure 1** illustrates the daily maximum temperatures in both heat-treated and controlled greenhouses in 2022-2023. The total duration of heat treatment was 77 days, with extreme temperatures above 40°C occurring on 24 and 55 days in the controlled and heat-treated greenhouses, respectively (**Figure 1**). The ventilation system was used to control set-point temperatures in both control and heat-treated greenhouses.

Before heat treatment, seeds from each accession were cultivated in a controlled greenhouse for approximately two months. The seeds of PHR18 were procured from the Vegetable Research Division, National Institute of Horticultural and Herbal Science (Wanju, Korea), while those of PHR23 came from Nongwoo Bio (Suwon, Korea). The seeds of two accessions were sown in plastic trays (54 × 28 cm in size, 6 × 12 cells with pot volume 2.4 L) that were filled with commercial bed soil (‘Bio Sangto’; Seoul, Korea) containing cocopeat (67.5%), peat moss (17%), zeolite (5%), per-lite (10.0%), pH adjuster (0.3%), humectant (0.014%), and fertilizers (0.185%) containing 270 mg kg^−1^ of each of N, P, and K, respectively. And then the seedlings were grown for 48 days in greenhouse (26/18°C in day/night (16/8 h) with relative humidity within 65–70%) until use. Seedlings were transplanted on May 3^rd^, 2022, and April 25^th^, 2023.

The experiment was laid out in split plot design with repeated measures across 2 years. The heat treatment is considered as a main plot, while accession is considered as a subplot. In each greenhouse, the subplot was laid out in a randomized complete block design with three replicates. In each replicate, 4 -5 plants were spaced 35 cm apart. The distance between single-row plots was 120 cm. During the treatment, plants were regularly fertigated using a drip irrigation system. The nutrient solutions of fertigation were A (N 5.5%, K 4.5%, Ca 4.5%, B 0.00014%, Fe 0.05%, Zn 0.0001%, and Mo 0.0002%) and B (N 6%, P 2%, K 4%, Mg 1%, B 0.05%, Mn 0.01%, Zn 0.005%, and Cu 0.0015%) (Mulpure, Daeyu, Seoul, Republic of Korea).The relative humidity in both greenhouses was between 50 and 85%. More details on the experimental setup and cultivars can be found in Kim et al. (2023).

#### 2.1.1 Collection of morphological traits and yields for two hot pepper genotypes

After heat treatment of the pepper crops PHR18 and PHR23, plant height, stem thickness, and width were measured using a ruler and a digital caliper (CD-20APX, Mitutoyo Co., Ltd., Kanagawa, Japan). In 2022, measurements were taken from 5 plants per replicate on days 1, 3, 5, 12, 26, 35, 42, 57, 75, and 91 after heat treatment; in 2023, measurements were from 4 plants per replicate on days 1, 3, 5, 7, 9, 13, 15, 17, 19, 21, 23, 25, 37, 49, 63, and 77. Three replicates were conducted. Plant height (cm) was recorded from the base to the top of the stem, stem thickness (mm) was measured at its lowest point, and plant width (cm) was determined by measuring the distance between the furthest points of the horizontally extended branches. For yield analysis, plants were harvested on days 35 and 75 in 2022, and on days 37 and 77 in 2023. One plant per replicate was harvested, and the fresh weight of leaves, stems, fruits, and flowers was measured. Leaf area was assessed using a leaf area meter (LICOR-300, Lincoln, NE, USA). After harvesting, samples were dried at 70°C, and the dry weights (g) of leaves, stems, fruits, and flowers were recorded to calculate plant water content and yield.

#### 2.1.2 Nitrogen analyses stems and fruit parts of pepper crops

Nitrogen content analysis was conducted using one plant per replicate, harvested on days 35^th^ and 75^th^ in 2022, and days 37^th^ and 77^th^ in 2023. The dried samples were ground, and 1 g of each sample (leaves, stems, fruits, and flowers) was placed into a 300 mL glass tube. The samples were digested using 15 mL of concentrated sulfuric acid (H_2_SO_4_) in a Kjeldahl digestion system (SH420F, Hanon, China). The digested samples were distilled using a distillation apparatus (K9840, Haineng Scientific Instrument Co., Ltd., Shandong, China), and 0.1N hydrochloric acid (HCl) was added gradually to measure the total nitrogen content.

The total N content (g/kg) can be calculated using the following equation:


$$\frac{\left(ml\;HCl_{sample}-ml\;HCl_{bland}\right)\times \left[HCl_{con}\right]\times 14.01\times 100)}{1000\times weight\;of\;samples\;\left(ɡ\right)\;\;}$$

### 2.2 Development of pepper-growth models

In this study, the process-based model ALMANAC (Agricultural Land Management Alternatives with Numerical Assessment Criteria) was used to simulate crop growth, partition biomass into fruits or grains, nutrient uptake, and address growth constraints such as water, temperature, and nutrient stress (Kiniry et al., 2002). This model, developed to simulate over 100 species, operates on a daily time step. Soil, weather, cropping schedule, and crop parameter are essential input for the model. This model also offers a wide range of tillage operations, including drainage, irrigation, fertilization, furrow diking, and liming. Thus, users can effectively run the model with various cropping management scenarios or different climate condition scenarios.

#### 2.2.1 Development of crop parameter sets of two pepper cultivars and model calibration

Some critical crop parameters in ALMANAC are outlined in **Table 1**. The ALMANAC model simulates LAI, light interception according to Beer’s law, and potential daily biomass increases, which are influenced by a species-specific radiation use efficiency (RUE). Under stress conditions (e.g., drought, flooding), the model accounts for reduced growth by decreasing daily increases in LAI and biomass (Kiniry et al., 2002). Plant development, calculated based on degree days, uses two temperature parameters: optimal temperature (TB) and base temperature (TG). The model considers over 50 plant parameters describing growth characteristics, including the fraction of nitrogen in the plant body. The experimental data described in Section 2.1 was used to obtain the parameters for different pepper cultivars grown under various environmental conditions.

**Table 1:** Some important crop parameters of two pepper accessions in control and heat-treated conditions.

Parameter	Description	PHR18		PHR23	
		Control	Heat	Control	Heat
WA	Potential growth rate per unit of intercepted photosynthetically active radiation	33	24	37	25
HI	Harvest index: Crop yield/above-ground biomass.	0.57	0.58	0.65	0.42
TB	Optimal temperature for plant growth	30	30	30	30
TG	Minimum temperature for plant growth	10	10	10	10
DMLA	Maximum leaf area index	3.8	3.8	6.1	6.1
DLAP1	Initial point on optimal leaf area development curve	10.19	10.19	10.19	10.19
DLAP2	Second point on optimal leaf area development curve	50.95	45.95	65.95	40.95
PPL1	Plant population parameter (Number before decimal represents plants m^-2^)	1.08	2.08	1.08	2.08
PPL2	Second plant population parameter	4.99	6.99	4.99	8.99
CNY	Normal fraction of N in yield	0.03	0.03	0.03	0.03
BN1	Normal fraction of N in crop biomass at emergence	0.03	0.03	0.03	0.03
BN2	Normal fraction of N in crop biomass at midseason	0.007	0.003	0.006	0.003
BN3	Normal fraction of N in crop biomass at maturity	0.003	0.001	0.003	0.001
PHU	Potential heat units from planting to physiological maturity	1800	3000	1800	3000

Based on the measured data, two cultivars exhibited different growth patterns. Additionally, the same cultivar showed varying responses to heat stress due to priming effects. Two plant parameters, LAP1 and LAP2, describe the plant growth curve during the growing season, varied by cultivar and growing conditions. The harvest index (HI) and maximum leaf area index (DMLA) were also derived from measured data. Plant canopy variation was influenced by environmental conditions. Under stressful conditions, the LAI decreased, leading to adjustments in two plant population parameters, PPL1 and PPL2, for heat-treated and controlled conditions. Parameters that describe nitrogen(N) use efficiency were also identified, including the normal fraction of N in total yield (CNY) and normal fractions of N at emergence (BN1), mid-season (BN2), and maturity (BN2). These parameters were determined by analyzing nitrogen yield in the plant body as described in Section 2.1.2.

To evaluate the plant parameters and test ALMANAC’s ability to accurately simulate different pepper cultivars in heat-treated and controlled environments, simulated fruit yields for 2022 and 2023 were compared with measured fruit yields from greenhouse field measurements. To test model goodness-of-fit, the percentage bias (PBISA), root-mean-square error (RMSE), and Pearson’s correlation coefficient of determination (R2) were calculated.

### 2.3 Calibration and validation of plant growth model in multiple locations in Korea

In Korea, over 90% of pepper production occurs in open fields. Accordingly, the developed model simulated pepper growth in various locations in South Korea. Since climatic conditions significantly influence plant development, it is essential to calculate the heat unit requirement for plant growth in specific locations. Heat units vary with different air temperatures, affecting ripening times for plants (Pramudia, 2021). For instance, heat accumulation in the southern regions of South Korea occurs more rapidly than in the northern regions due to warmer air temperatures. The developed pepper model simulated pepper yield across multiple locations in South Korea. By comparing simulated and measured yields across multiple locations and years, the heat units for all locations were established. For the simulations, a set of plant parameters describing the pepper of PHR18 accession grown in controlled conditions was used, as the morphological characteristics of PHR18 are similar to those of accessions grown in field sites.

Radiation use efficiency (RUE) of plants grown in open fields is lower than that of plants in greenhouses because the ability to control environmental conditions such as temperature, wind, or CO_2_ in open fields is limited (Jin et al., 2023). Consequently, to adjust the value of WA in open fields, a study was conducted on a small farm located in Cheonan city, Chungcheongnam province, South Korea (36°46′ N, 127°07′ E). One-month-old seedling plants of the commercial cultivar ‘Callatan’ were transplanted on April 7^th^, 2024. The plants were spaced at 35 cm intervals, with a distance of 100 cm between single-row plots. All fertilization and general cultivation practices adhered to the ‘Standard Farming Manual’ (RDA). Two harvests were carried out on the day of the 107^th^ (July 22^nd^) and 130th day (August 14^th^) after transplanting. The total yield was determined by summing the yields of the two fruit harvests. The harvested samples were dried in a 70°C oven until a constant mass was achieved. Field data collected was utilized to estimate the field WA (Biomass-Energy ratio) value in simulations.

**Figure 2:**
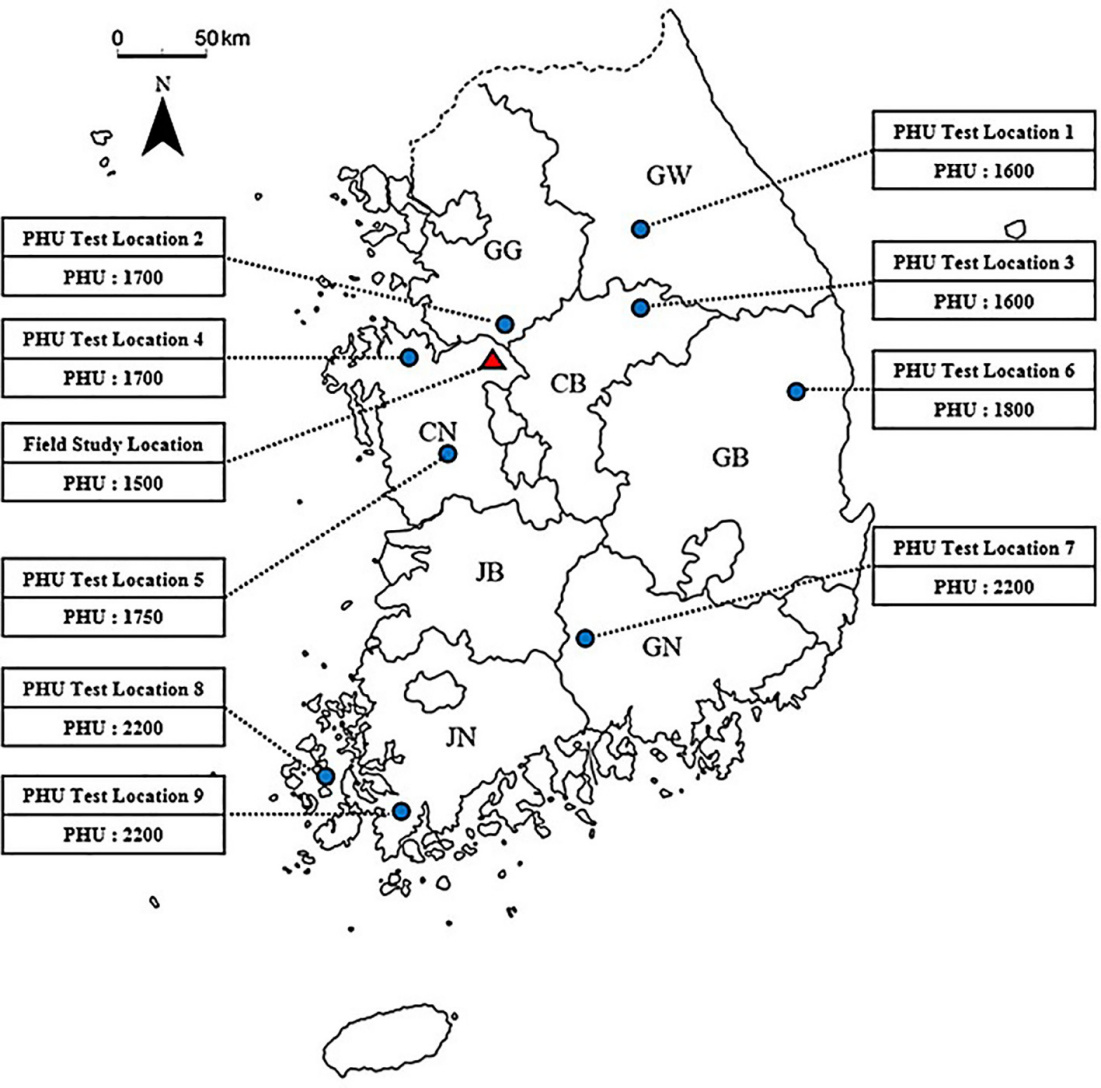
A field study site along with 9 PHU test field sites across 7 provinces in South Korea, marked on the map. The box numbers represent the estimated potential heat units (PHU) for each site. The map abbreviations for the provinces are as follows: GW, Gangwon; GG, Gyeonggi; CN, Chungcheongnam; CB, Chungcheongbuk; GB, Gyeongsangbuk; GN, Gyeongsangnam; JB, Jeollabuk; JN, Jeollanam.

To determine PHU in various locations in South Korea, yield data of various commercial pepper varieties from 2020-2023 was gathered from 9 field sites across 7 provinces for comparison with simulated yields. Each province featured multiple field sites, and the PHU values varied by location (**Figure 2**). All field data were collected by the Korea Rural Economic Institute (KREI). Seeds were sown in greenhouse pots at varying times depending on the region and were transplanted to the open field from early April to mid-May, based on the optimal planting timing for each area. The seedlings were transplanted at a spacing of 50 cm × 40 cm, with a plant density of about 2-3 plants per square meter. Crop management conformed to the standard agricultural practices of the Rural Development Administration (RDA). Growth observations were conducted from 1 to 9 times at intervals of 2-3 weeks, starting about a month post-transplantation. The measured fruit yields were calculated by summing up the yields of fruits harvested 5-6 times throughout the growing period. The final harvest occurred between late August and early October. Unlike the greenhouse experiment, crops in the field were harvested when their leaf area began to decline, thus setting the DLAI (fraction of the growing season when the leaf area declines) at 0.9 for open field simulations. Yields severely affected by insects, diseases, or other factors were excluded from the yield comparisons.

To test model goodness-of-fit, the percentage bias (PBISA), root-mean-square error (RMSE), and Pearson’s correlation coefficient of determination (R2) were calculated. And the relative ratio between simulated and measured fall biomass productions was calculated for site.

### 2.4 Evaluation of heat stress effects on pepper yields in South Korea

After successfully calibrating the field simulation, it was employed to assess the impacts of elevated temperatures on the yields of two pepper varieties (PHR18 and PHR23) at multiple locations throughout South Korea. A total of 9 field sites from 7 provinces were used for simulation. Yield variations were modeled for scenarios where the maximum and minimum temperatures increased by 3-5°C. Historical weather data spanning 2014-2023 served as the basis for reference yields, and the crop parameter sets developed in Section 2.3 were applied to reference simulations. As per the greenhouse experiment detailed in Section 2.1, the temperature differential between control and heat-stressed conditions ranged from 3-5°C. Consequently, crop parameter sets for PHR18 and PHR23 were utilized to simulate crop growth under high temperatures. Adjustments were made to certain crop parameters (WA and DLAI) and PHU as the crops were cultivated at field sites (refer to Section 2.1). For high temperature simulations, additions of 3-5°C were applied to both maximum and minimum temperatures. Other inputs such as cropping management and soil conditions remained consistent with reference scenarios.

## 3 Results

### 3.1 Determination of pepper growth parameters under heat stress conditions

To determine crop growth parameters and the growth curve of pepper accessions under various conditions, 2-year greenhouse studies were conducted. Yield and morphological characteristics of different pepper accessions were monitored after exposure to prolonged heat stress (**Tables 2**, **3**). Statistical analysis for all variables is presented in **Table 4**. The yields of both accessions were significantly affected by heat stress (*P*=0.0253). The two pepper accessions exhibited different growth patterns under heat stress (*P*=0.0021). During the first 35^th^ days of heat treatment, PHR18 exhibited significant yield losses in total weight (-45%) and fruits (-34%), whereas PHR23 showed increases in both total weight (+18%) and fruits (+64%). With prolonged heat stress, both accessions experienced significant yield reductions in total weight and fruits. However, lesser yield losses were observed in PHR18 by the 75^th^ day of heat treatment. PHR23 yielded higher biomass and fruit than PHR18. The harvest index values for PHR23 were higher than those for PHR18 in control conditions on both the 35^th^ and 75^th^ days of heat treatment (*P*=0.0056). On the 35th day of heat treatment, both accessions had a higher harvest index in heat-treated conditions compared to control conditions, while the harvest indexes were similar in both conditions on the 75^th^ day of treatment (*P*=0.0052).

**Table 2:** Total fresh weight, fruit weight, and harvest index of PHR18 and PHR23 accessions under control and heat stress conditions.

Days after heat treatment	Lines	Control condition			Heat stress condition			Yield difference (%)	
		Total fresh mass (g)	Fresh fruit mass (g)	Harvest index	Total fresh mass (g)	Fresh fruit mass (g)	Harvest index	Total mass	Fruit mass
35 days	PHR18	326 ± 108	91 ± 51	0.28	178 ± 123	60 ± 36	0.34	-45	-34
	PHR23	803 ± 180	312 ± 105	0.39	950 ± 507	513 ± 286	0.54	18	64
75 days	PHR18	1508 ± 314	825 ± 150	0.55	980 ± 290	565 ± 228	0.58	-35	-32
	PHR23	2002 ± 401	1303 ± 277	0.65	1560 ± 208	846 ± 152	0.54	-22	-35

Yield differences were calculated by comparing the average yields between control and heat stress conditions across the 2022 and 2023 seasons.

**Table 3:** Morphological characteristics and total nitrogen (g) of two pepper accessions (PHR18 and PHR23) in control and heat treatment conditions across 35 and 75 days in 2022 -2023.

Treatment	35 days post-heat stress						
	Accessions	No. of fruits/plant	Moisture content (%)	Leaf area index (LAI)	Height (cm)	Stem thickness (mm)	Total N (g)
Control	PHR18	38 ± 18	87 ± 2	1.51 ± 0.48	72 ± 9	13.62 ± 1.42	0.18 ± 0.05
	PHR23	54 ± 12	85 ± 1	4.01 ± 0.62	86 ± 13	16.12 ± 2.27	0.44 ± 0.13
Heat	PHR18	28 ± 15	77 ± 22	0.72 ± 0.61	59 ± 18	10.74 ± 2.84	0.10 ± 0.05
	PHR23	52 ± 18	87 ± 2	3.22 ± 2.14	81 ± 27	15.35 ± 4.30	0.49 ± 0.30
75 days post-heat stress							
Control	PHR18	119 ± 54	88 ± 4	2.71 ± 0.37	123 ± 12	16.28 ± 1.94	0.54 ± 0.11
	PHR23	65 ± 23	86 ± 1	4.12 ± 0.95	128 ± 13	19.23 ± 3.17	0.80 ± 0.23
Heat	PHR18	94 ± 52	86 ± 2	2.10 ± 0.64	102 ± 27	13.41 ± 2.47	0.43 ± 0.17
	PHR23	47 ± 3	87 ± 3	2.71 ± 0.78	105 ± 33	19.91 ± 6.02	0.58 ± 0.14

**Table 4:** Statistical analysis (ANOVA) for evaluating the significance of yield, morphological characteristics, and total nitrogen (Total N) of two accessions (PHR18 and PHR23) grown in control and heat stress conditions in 2022-2023.

Variable	Yield	Harvest index	Number of fruits	Moisture content	Leaf area Index	Height	Stem thickness	Total N
Treatment (Trt)	0.0253	n.s.	n.s.	n.s.	0.0002	0.0026	0.0023	0.0242
Harvest days (Days)	<0.0001	<0.0001	0.0013	n.s.	0.0217	<0.0001	<0.0001	<0.0001
Accession (AC)	0.0021	0.0056	n.s.	n.s.	<0.0001	n.s.	<0.0001	<0.0001
Trt*days	0.0026	0.0052	n.s.	n.s.	n.s.	n.s.	n.s.	0.0295
Trt*AC	n.s.	n.s.	n.s.	n.s.	n.s.	n.s.	n.s.	n.s.
Days*AC	n.s.	n.s.	0.0081	n.s.	0.006	n.s.	n.s.	n.s.
Trt*Days*AC	n.s.	n.s.	n.s.	n.s.	n.s.	n.s.	n.s.	n.s.

n.s. indicates no significant difference at alpha=0.05.

On the 75th day of heat treatment, PHR18 exhibited a higher fruit count than PHR23 under both control and heat treatment conditions, indicating that PHR23 produced larger fruits but in lower quantities than PHR18 (**Table 3**, *P*=0.0081). During the initial 35 days of heat treatment, while water content showed no significant differences, PHR18’s water content at 77% was considerably lower than the control’s 87%. By the 35^th^ day, the leaf area of PHR18 had halved compared to the control, whereas PHR23’s leaf area decreased by about 18% under heat stress conditions (*P*=0.0006). After 75^th^ days, the leaf area reduction of PHR18 was approximately 22% in heat treatment conditions, while PHR23’s leaf area halved compared to the control. PHR23 exhibited greater height and stem thickness than PHR18. Both accessions presented reduced heights (*P*=0.0026) and smaller stem thickness (*P*=0.0023) under heat treatment conditions (**Table 3**). The total nitrogen content in the biomass and fruits of both accessions varied across different harvest times and conditions (**Table 3**). Lower total nitrogen levels were observed in the heat stress condition compared to the control (*P*=0.0242). PHR23 consistently showed higher total nitrogen content than PHR18 across all days and conditions, with significant differences (P<0.0001). Notably, on the 75^th^ day of treatment, more substantial reductions in total nitrogen were noted (*P*=0.0295).

**Figure 3:**
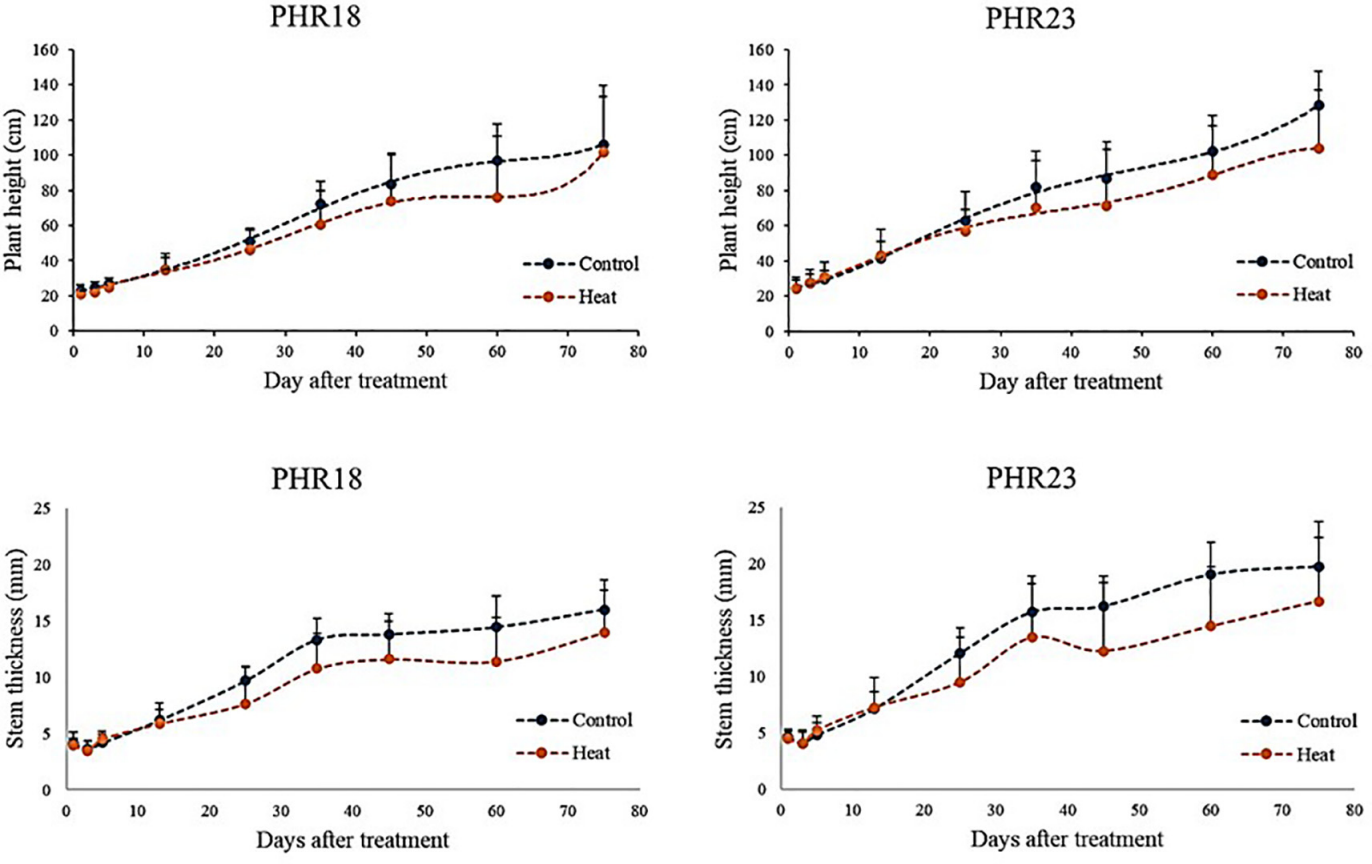
Plant height and stem thickness of two pepper accessions, PHR18 and PHR23, in control and heat stress conditions in 2022 and 2023.

Over 75 days post-heat exposure, the plant height and stem thickness of both accessions were monitored (**Figure 3**). For the first 10 days, there were no differences in height and stem thickness between control and heat stress conditions. However, after 10^th^ day, both accessions began to show a reduction in growth and biomass accumulation (**Figure 3**). Despite being in heat treatment conditions, growth continued until the experiments concluded. By the 75^th^ day of heat treatment, PHR18’s height closely approximated that in the control condition.

### 3.2 Development of crop parameters of two pepper accession in control and heat stress conditions

Based on field data, key crop parameters were determined. The sets of parameters used in the simulation are listed in **Table 1**. Observations of growth characteristics showed that PHR23 exhibited greater biomass and fruit yields than PHR18. Consequently, the HI values for PHR18 and PHR23 in control conditions were 0.57 and 0.65, respectively, chosen from the highest observations within each accession. Additionally, the maximum potential leaf area index, DMLA, for PHR23 was higher than that for PHR18. Growth curve parameters, DLAP1 and DLAP2, were established based on measured values. For PHR18, DLAP1 and DLAP2 were 10.18 and 50.95, respectively, while for PHR23, they were 10.19 and 65.95, respectively. The BN2 and BN3 values were calculated from the total N yields of the biomass for both accessions. BN2 and BN3 for PHR18 and PHR23 were approximately 0.006 and 0.003, respectively. WA and PHU were defined during model calibration. Under heat stress conditions, plant growth was slower than under control conditions. Thus, potential heat units in heat stress conditions were longer than in control conditions, and the potential growth rate, WA, was lower in heat stress conditions. Biomass components such as leaf area, height, and stem thickness were significantly reduced under heat stress (**Table 3**). The DMLA values were consistent across both conditions, but the values of PPL1 and PPL2 were adjusted (**Table 1**).

**Figure 4:**
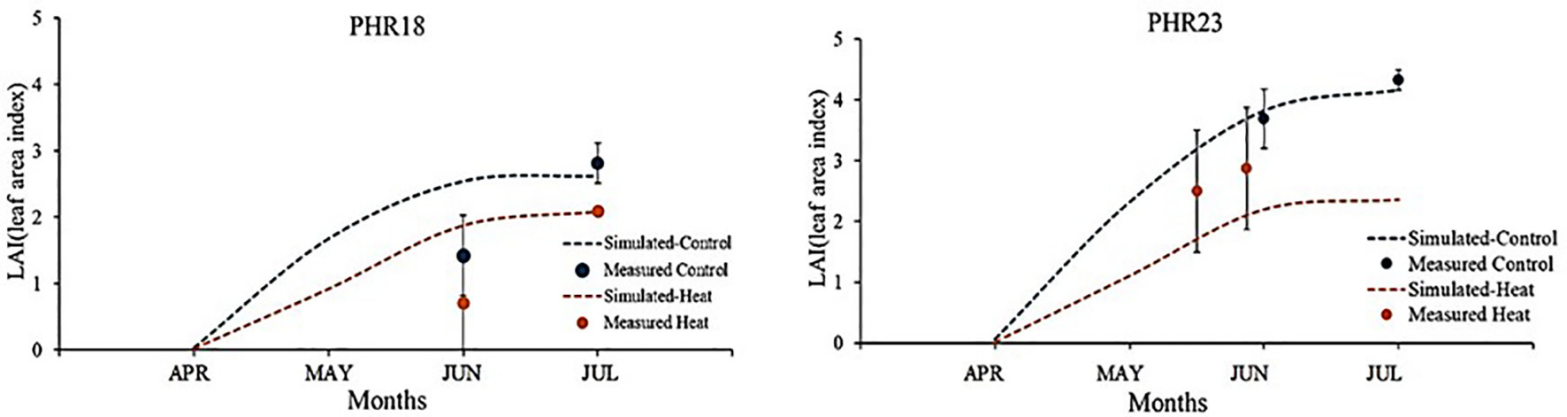
Comparison of measured and simulated leaf area index (LAI) of two pepper accessions, PHR18 and PHR23, grown in control and heat conditions during 2022-2023.

After developing parameter sets and plant growth curves, the pepper growth model was calibrated using measured LAI and yields. Simulated and measured leaf area growths were compared (**Figure 4**). Under control conditions, the measured leaf area index of PHR23 closely matched the simulated leaf area curve. However, under heat stress conditions, the simulated LAI was higher than the measured LAI growth. This discrepancy might be due to large variations in LAI observed within each accession under heat stress. Although the measured LAI under heat stress did not align with the simulated LAI growth curve, the simulated curve fell within the range of measured LAI variation. For PHR18, in both control and heat stress conditions, the simulated LAI at the 75^th^ day closely matched the measured LAI, while the simulated growth at the 35^th^ day was overestimated (**Figure 4**). In 2023, the yields were significantly impacted by viruses (e.g., Cucumber mosaic virus), leading to substantial reductions in yields and LAI.

**Figure 5:**
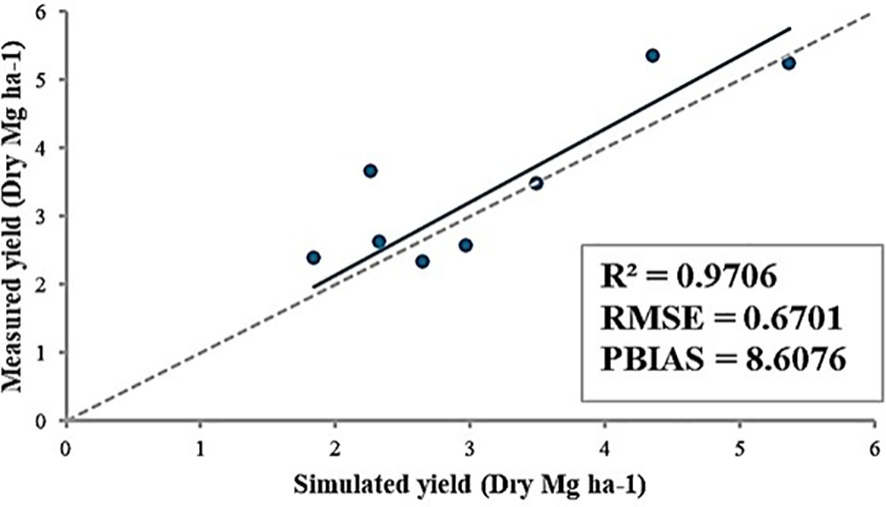
Comparison between measured and simulated yields of PHR18 and PHR23 under control and heat stress conditions for 2022-2023. Calculations for R^2^, RMSE, and PBIAS are also presented.

Overall, the model was successfully developed (**Figure 5**, R^2^ = 0.97). In control conditions, the yields of PHR18 for 2022 and 2023 were measured at 3.49 and 2.26 Mg ha^-1^, respectively; the simulations yielded 3.47 and 3.66 Mg ha^-1^. Under heat stress conditions, the yields were 1.84 and 2.33 Mg ha^-1^ for the same years, while simulations showed 2.38 and 2.63 Mg ha^-1^. For PHR23 under control conditions, measured yields for 2022 and 2023 were 5.37 and 4.36 Mg ha^-1^, whereas the simulations were slightly lower at 5.25 and 5.35 Mg ha^-1^, respectively. Under heat stress conditions, measured yields were 2.65 and 2.97 Mg ha^-1^ respectively, with simulated yields at 2.34 and 2.57 Mg ha^-1^. The calculated RMSE and percentage of bias (PBIAS) were 0.94 Mg ha^-1^ and 8.6%, respectively (**Figure 5**). Simulation results indicate that the ALMANAC model tended to slightly overestimate yields.

### 3.3 Determine potential heat units of pepper across multiple locations through simulation

A field study in Cheonan city estimated the WA of pepper in an open field. Results indicated that the radiation use efficiency of pepper was higher in the greenhouse than in the open field. The greenhouse WA for PHR18 was 33, while the simulation for the open field was 27. The model successfully simulated the field sites, as evidenced by the yield ratio between measured and simulated values being 1.01 (**Table 5**). Thus, the simulated yield of pepper was 8.05 Mg ha^-1^, compared to the measured yield of 8.13 Mg ha^-1^ at the field site (**Table 5**).

**Table 5:** Comparison between simulated and measured yields at a field study site and 9 PHU test locations in South Korea.

Simulation sites	Province	City	Year	Measured yield (Dry Mg ha-1)	Simulated yield (Dry Mg ha-1)	Measured/ Simulated
Field Study Location	CN	Cheonan	2024*	8.13 ± 1.77	8.05	1.01
PHU Test Location 1	GW	Hoengseong	2021*	8.23 ± 0.54	7.81	1.05
PHU Test Location 2	GG	Anseong	2021*	9.01 ± 1.48	8.18	1.10
			2023*	8.89 ± 1.73	7.30	1.22
PHU Test Location 3	CB	Jecheon	2021*	8.57 ± 2.78	9.42	0.91
			2022*	7.87 ± 0.19	8.49	0.93
			2023*	8.93 ± 0.00	9.46	0.94
PHU Test Location 4	CN	Dangjin	2021*	7.16 ± 2.27	7.79	0.92
PHU Test Location 5	CN	Cheongyang	2021*	6.17 ± 0.09	7.26	0.85
			2022*	7.68 ± 0.82	8.66	0.89
PHU Test Location 6	GB	Yeongyang	2021*	7.83 ± 1.47	7.70	1.02
			2022*	7.07 ± 1.11	7.30	0.97
			2023*	8.39 ± 0.31	9.49	0.88
PHU Test Location 7	GN	Hamyang	2021*	11.71 ± 2.02	11.18	1.05
			2023*	11.28 ± 1.69	11.16	1.01
PHU Test Location 8	JN	Sinan	2021*	10.85 ± 1.18	10.59	1.02
PHU Test Location 9	JN	Haenam	2021*	7.46 ± 2.61	7.43	1.00

The ratios of measured to simulated yields are listed in the table.

**Figure 6:**
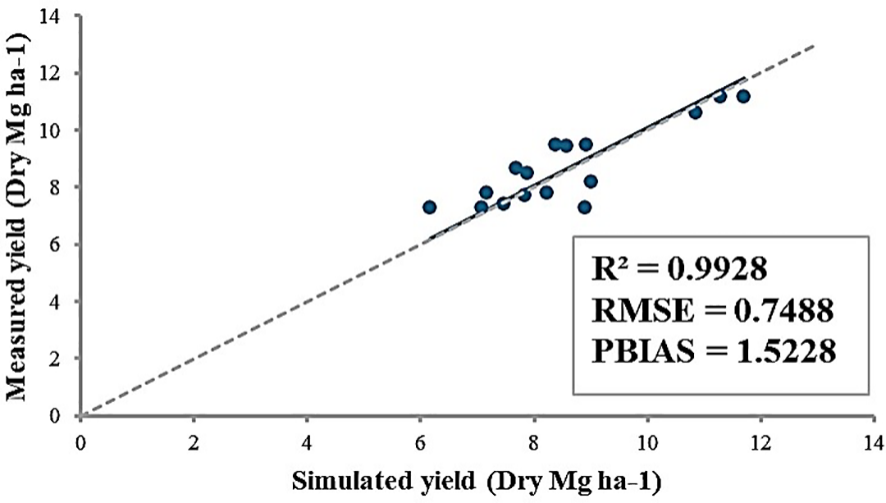
Comparison of measured and simulated yields of pepper from 9 field sites in South Korea between 2021 and 2024. The R^2^, RMSE, and PBIAS were calculated.

After adjusting the WA parameter for an open field, 9 field sites were selected to estimate potential heat units (PHU) at each location (**Table 5**). The estimated PHU is depicted in **Figure 2**. According to simulation results (**Table 5**), high PHUs were observed in the southern parts of South Korea. In the northeastern region of South Korea, lower PHUs (1500) were recorded, while PHUs in the west to central regions ranged from 1700-1800. In the northeastern regions, nearly 80% of the area consists of mountains, resulting in average temperatures that are comparatively lower than in other regions. The southern regions of South Korea experienced warmer, milder weather compared to other regions. Overall, the model effectively simulated the yields at 9 field sites, achieving an R^2^ = 0.99. RMSE and percent bias (PBIAS) calculations were 0.75 Mg ha^-1^ and 1.5%, respectively (**Figure 6**). The average measured yield of pepper across all regions was approximately 8.56 Mg ha^-1^, while the simulated average yield was 8.7 Mg ha^-1^. Yields in the southern regions exceeded those in other regions. At locations 7-8, yields surpassed 11 Mg ha^-1^ (**Table 5**).

### 3.4 Impacts of high temperatures on pepper yields across various locations in South Korea

Significant yield reductions in PHR18 and PHR23 were observed when temperatures increased by 3-5°C (**Table 6**). In most locations, the extent of yield reduction incrementally increased as temperatures rose from 3°C to 5°C (**Table 6**). PHR23 experienced greater yield reductions than PHR18. For PHR18, yields decreased by 31-44% from the reference yield, while PHR23 saw reductions up to 53%. Yield reductions also varied by location. In Anseong city, located in the northern region of South Korea, the smallest reductions were observed for both pepper varieties. This was attributed to the shorter potential heat units (PHU) in the northern region compared to others (**Figure 2**), resulting in similar timing of maximum PHU in reference and heat stress conditions. In contrast, the greatest yield reductions occurred in the southern region of Korea, for example in Haename city. Simulation results indicate that under heat stress conditions, the maximum heat units occurred approximately two months earlier than in the reference years. For instance, plants continued to grow until October in reference years but ceased growth in August under heat stress conditions. Additionally, more irrigation was required under heat stress than in reference conditions, indicating that water stress was also a factor (data not shown). Heat stress adversely affected plant growth and development, leading to reduced potential growth rates, WA, for plants under these conditions (**Table 1**), which significantly contributed to the yield reductions.

**Table 6:** Simulated yields of PHR18(A) and PHR23(B) peppers for the reference years 2014-2023, and at increased temperatures of 3°, 4°, and 5°C, across various locations in South Korea.

A. Simulated yields of PHR18 in multiple locations in South Korea.					
Province	City	Reference yield (Dry Mg ha^-1^)	+3°CSimulated yield (Dry Mg ha^-1^)	+4°CSimulated yield (Dry Mg ha^-1^)	+5°CSimulated yield (Dry Mg ha^-1^)
GW	Hoengseong	7.53	4.88(-35)	4.85(-36)	4.82(-36)
GG	Anseong	8.13	5.57(-31)	5.58(-31)	5.54(-32)
CB	Jecheon	9.04	5.99(-34)	6.15(-32)	6.04(-33)
CN	Dangjin	10.61	6.12(-42)	5.95(-44)	5.94(-44)
	Cheongyang	9.58	5.56(-42)	5.45(-43)	5.37(-44)
GB	Yeongyang	9.21	6.20(-33)	6.21(-33)	6.21(-33)
GN	Hamyang	11.16	7.24(-35)	7.18(-36)	7.14(-36)
JN	Sinan	11.17	6.69(-40)	6.63(-41)	6.56(-41)
	Haenam	10.92	6.20(-43)	6.41(-41)	5.99(-45)
B. Simulated yields of PHR23 in multiple locations in South Korea.					
Province	City	Reference yield (Dry Mg ha-1)	+3°C Simulated yield (Dry Mg ha-1)	+4°C Simulated yield (Dry Mg ha-1)	+5°C Simulated yield (Dry Mg ha-1)
GW	Hoengseong	9.36	4.97(-47)	4.93(-47)	4.88(-48)
GG	Anseong	10.12	5.65(-44)	5.65(-44)	5.61(-45)
CB	Jecheon	11.15	6.15(-45)	6.20(-44)	6.15(-45)
CN	Dangjin	13.17	6.24(-53)	6.10(-54)	6.09(-54)
	Cheongyang	11.90	5.83(-51)	5.71(-52)	5.62(-53)
GB	Yeongyang	11.48	6.31(-45)	6.31(-45)	6.29(-45)
GN	Hamyang	13.84	7.37(-47)	7.31(-47)	7.27(-48)
JN	Sinan	13.90	6.94(-50)	6.87(-51)	6.80(-51)
	Haenam	13.39	6.33(-53)	6.50(-51)	6.41(-52)

The figures for parentheses show the percentage yield loss compared to reference yields. Abbreviations for provinces on the map are GW, Gangwon; GG, Gyeonggi; CN, Chungcheongnam; CB, Chungcheongbuk; GB, Gyeongsangbuk; GN, Gyeongsangnam; JB, Jeollabuk; JN, Jeollanam.

## 4 Discussion

The impact of climate change, manifesting as increased variability such as heat and droughts, has adversely affected crop growth and development. Although pepper thrives in warm seasons, yields can significantly decline when temperatures exceed 35°C during hot summers (Zakir et al., 2024). Weather reports from the Korea Meteorological Administration (KMA, 2024) indicate that average summer temperatures have risen by 2.8°C. Additionally, the frequency of heat waves, defined as days with maximum temperatures over 33°C, was 2.3 times higher than in typical years. Unanticipated heat stress events have led to an anticipated 10-15% reduction in pepper yields for 2024. The increased vulnerability of pepper yields is a direct result of more frequent unexpected weather events. The development of a pepper yield prediction model under extreme weather conditions for various regions has become increasingly crucial for farmers and policymakers in South Korea. To optimize the pepper yield prediction under heat stress conditions, this paper presents a study conducted over 2 years in a greenhouse and 1 year in the field, aimed at developing the plant growth curve and crop parameters.

There are not many pepper simulation studies that have investigated the effects of heat stress on pepper yields, primarily because few studies have monitored pepper yields under prolonged heat stress conditions (Kim et al., 2023). Although numerous studies have explored the impact of heat stress on pepper growth, development, and reproductive behavior (Saha et al., 2010; Utami and Aryanti, 2021; Aruna et al., 2023), most have only examined physiological and genetic changes after brief periods of heat exposure, ranging from hours to weeks. The limited understanding of physiological changes or crop growth behavior after prolonged heat stress exposures can hinder the development of a yield prediction model under such conditions. In this study, two genotypes, PHR18 and PHR23, responded differently to heat stress. The PHR23 accession tends to allocate more resources to biomass, such as stems and leaves, rather than reproductive parts like fruits, whereas the PHR18 accession prioritizes fruit production over biomass. Thus, two different growth curves for PHR18 and PHR23 were developed in the model.

Unlike PHR18, the PHR23 accession, which has a larger leaf area, showed positive impacts on yield during the first month of heat stress exposure, suggesting that leaf or canopy number may play a role in thermal tolerance. The PHR23 accession’s larger leaves, which house more chloroplasts, can capture more sunlight and enhance photosynthesis rates under elevated temperatures, potentially increasing yields (Hu et al., 2020). However, after a month of heat stress, PHR23 began to exhibit reduced growth and development. Yield reductions of 22 and 35% were observed in PHR23’s total biomass and fruits, respectively. PHR18 exhibited stress priming after a month of heat stress, resulting in a lesser yield reduction in the second harvest (75 days after heat stress). Based on these observation, the plant parameters including WA, DLAP1 and 2, and PPL1 and 2, were determined. The pepper model accurately simulated yields under control conditions; however, it could not effectively predict yields under heat stress conditions when plants sustained more damage from disease. Under heat stress, in the experiment, plants suffered from both heat stress and diseases. Higher temperatures often exacerbate the severity of plant diseases (Cohen and Leach, 2020), potentially leading to yield reductions. To enhance the accuracy of yield predictions for crops growing at higher temperatures, the simulation must recognize that plants can experience multiple stresses concurrently. However, responses to combinations of stress are complex and difficult to predict (Gupta and Senthil-Kumar, 2017), necessitating additional experimental data.

After developing crop parameter sets for two different pepper accessions in varied environmental conditions, key parameters such as PHU and WA were adjusted to predict pepper yield in open fields. Given that most peppers in Korea are cultivated in open fields, this model calibration is essential for generating more realistic outputs. A field study was conducted to compare simulated yields, and field survey data from various locations in South Korea were used to determine PHUs at different simulation sites. Since temperatures vary by location, it is crucial to determine the maximum heat units for peppers. In the southern regions of Korea, the PHU was higher due to more elevated daily temperatures, whereas in the northern and central regions, the PHUs ranged from 1500 to 1700. After successfully calibrating models for open-field simulations, we explored the effects of heat stress on pepper yields across various South Korean locations. Overall, the yields of both pepper accessions were reduced by approximately 31-53% from reference yields. As temperatures increased, plants reached their maximum potential heat units sooner than in control conditions, and growth rates of plants under heat stress were slower than those in control conditions. These factors primarily caused yield reductions at higher temperatures. The results can be supported by previous studies (Porter, 2005; Liu et al., 2010; Hatfield et al., 2011). Higher temperatures can accelerate accumulation of growing degree days, or heat units, resulting in shorter growing season. This would lead to reduction in light interception during growing season, resulting in a decrease in crop production. Additionally, a shorter growing season could cause an inefficient use of water and nutrients, which also plays a critical role in yield reduction under heat stress condition (Harrison et al., 2011). In simulation, increased irrigation was required under heat stress conditions, indicating that plants also suffered from drought stress. Based on the simulation results, PHR18 was more resilient under heat stress conditions than PHR23, as evidenced by smaller yield reductions observed at most simulation sites. Moreover, the nitrogen stress days when plants are deficient in nitrogen increased as the temperature increased in simulation.

As climate change has significantly increased attention, many studies use crop models to assess the impact of climate change on crop yields. However, most studies examined how the same crop parameter sets performed under different climate conditions (Mangani et al., 2023; Zare et al., 2023). They often missed the physiological and morphological changes in crops when they were exposed to heat stress. This study may be the first attempt to develop pepper growth model based on experimental data. The developed model was able to illustrate two pepper growth patterns under heat stress condition. However, although the rates of yield reduction varied by genotypes, only two genotype parameter sets were developed in this study. This can increase uncertainty in the model. Since pepper is an economically significant fruit-bearing vegetable in Korea, numerous pepper accessions (over 300) from diverse genetic backgrounds have been developed, and over 100 commercial varieties are cultivated across various South Korean locations. Future studies could focus on determining optimal cropping management strategies under heat stress conditions by further investigating the impact of heat stress on multiple accessions. Using field data, plant parameter sets for various accessions can be developed and used to select the most appropriate pepper variety that can sustain high yields and production under heat stress conditions.

## 5 Conclusion

In this study, the ALMANAC model was used to explore the role of heat stress on pepper yields at multiple sites in South Korea. The negative effects of heat stress on pepper yields are well-known among scientists, and with the increasing frequency of heat waves due to climate change, there has been a significant reduction in pepper yields. However, developing a crop growth model under stressful conditions has been severely limited by the lack of field data. To enhance predictions of crop production under heat stress conditions, it is necessary to conduct field studies that assess the impact of long-term heat stress on plant growth. This study was the first attempt to simulate pepper production under heat stress conditions. After appropriate parameter calibration, ALMANAC model was capable of simulating pepper yields under heat stress conditions. The percent bias was under 8.6% and R^2^ was 0.97, indicating decent model performance in yield estimation under stressful conditions. The developed model was used to evaluate the impacts of climate change on pepper yields in multiple locations in South Korea. The study revealed that rising temperatures had negative effects on pepper yields shown as yields were reduced by 30 -50 percent from the reference yields. According to simulation results, plants reached their maximum potential heat unit fast under heat stress, resulting in yield reduction. This knowledge can inform decision-making on crop management and assist policymakers in maintaining price stability at the consumer level. This will help farmers mitigate profit loss.

## Data Availability

The raw data supporting the conclusions of this article will be made available by the authors, without undue reservation.
